# Gold nanoparticles disrupt actin organization and pulmonary endothelial barriers

**DOI:** 10.1038/s41598-020-70148-1

**Published:** 2020-08-07

**Authors:** Whitney E. Sinclair, Huei-Huei Chang, Arkaprava Dan, Paul J. A. Kenis, Catherine J. Murphy, Deborah E. Leckband

**Affiliations:** 1 Department of Chemical and Biomolecular Engineering, University of Illinois at Urbana-Champaign, Urbana, 61801 USA; 2 Department of Chemistry, University of Illinois at Urbana-Champaign, Urbana, 61801 USA; 3 Carl Woese Institute for Genomic Biology, University of Illinois at Urbana-Champaign, Urbana, 61801 USA

**Keywords:** Cellular imaging, Cytoskeleton, Actin, Nanoparticles, Confocal microscopy

## Abstract

This study explored the impact of gold nanoparticles on the metabolic activity and morphology of human pulmonary endothelial cell monolayers. We developed a gold nanoparticle library of three different sizes and two surface chemistries that include anionic citrate and the cationic polyelectrolyte poly(allylamine hydrochloride). The nanoparticles were characterized in cell culture medium to assess how their physical properties are altered after exposure to biological fluids. A bovine serum albumin pretreatment protocol was developed to stabilize the nanoparticles in cell culture medium. Results of this study show that an 18 h exposure of human pulmonary artery endothelial cells to the different nanoparticles modestly affects cellular metabolic activity. However, nanoparticle exposure perturbs the cortical actin networks and induces the formation of intercellular gaps. In particular, exposure to the poly(allylamine hydrochloride)-coated particles reduces the area of cell–cell junctions—a change that correlates with increased leakiness of endothelial barriers. The presence of excess polyelectrolyte capping agents in the supernatant of poly(allylamine hydrochloride)-coated nanoparticles significantly impacts endothelial morphology. Pretreatment of the particle supernatant with bovine serum albumin mitigates the negative effects of free or bound polyelectrolytes on endothelial cell monolayers.

## Introduction

The increased use of nanoparticles (NPs) in a wide range of applications including antimicrobial agents^1,2^, bioremediation^3,4^, fuel cells^5,6^, and biomedical treatments^7^ raises concern regarding their potential impact on human health. Many applications of NPs are already in the clinic, including drug carriers, imaging agents, and photothermal therapeutics^8–10^. Outside of these applications, NPs in the environment are a subset of particulate matter (PM) with a diameter of less than 2.5 microm (PM 2.5), and have shown adverse effects on human health, especially in the lung^11–13^. Following inhalation, these NPs can penetrate into lung tissue, at times causing inflammation and oxidative stress^14,15^.

Whether NPs enter the body through inhalation or intravenous administration, the circulatory system will be one of the first barriers with which NPs interact^16^. Endothelial cells that line the vasculature not only act as a barrier between blood and the surrounding tissue, but also restrict entry of NPs into the bloodstream. Specifically, lung alveoli are surrounded by vascular endothelial cells, and NPs must cross this bilayer of cells to enter the circulatory system. Several prior studies have reported observations regarding the effect of NPs on cell biology. Lin et al. highlights that NPs interrupt the epithelial cellular barrier by disrupting intercellular junctions^17^. Ma et al. reported endothelial cell morphology changes that are induced by NP treatment^18^. Liu et al. concluded that changes in actin morphology following NP treatment result in endothelial barrier dysfunction^19^. In addition, NP induced morphological changes correlated with increased vascular leakiness have been reported by Setyawati et al.^20^ Endothelial cell–cell junctions require vascular endothelial (VE)-cadherin adhesion molecules linked to the actin cytoskeleton of each cell^21^. Disruption of these VE-cadherin junctions results in leakage across this barrier. Recent studies focused on the impact of NPs on endothelial barrier leakiness due to the disruption of these VE-cadherin interations^22^. The NP induced leakage is a possible route for undesired paracellular transport of inhaled NPs to the circulatory system. Moreover, others have designed NPs as tools to facilitate endothelial leakiness for controlled drug delivery^23^. In contrast, work reported here focuses on the potential impact that environmental exposure to NPs can have on human tissue.

Importantly, exposure to biological fluids alters the physical properties of NPs, due to the adsorption of proteins and other biological macromolecules present in biological fluids^24^. The NP aggregation state can be altered in the presence of biomolecules – for instance, proteins have been found to stabilize colloidal NPs, while protein-free biological fluids induce NP aggregation^25^. Linse et al. defined the presentation of proteins on a particle surface as the protein corona^26^. While coating NPs with a protein corona can lead to off target or undesired biological responses, Chen et al. were the first to develop a pre-formed protein corona to achieve safe biomedical and environmental effects of ZnO NPs^27–29^. Therefore, developing processing protocols for maintaining NP colloidal stability in complex biological media is desirable.

To reveal the environmental impact of NPs on the human lung, we report the acute impact of gold nanoparticles (AuNPs) on the metabolic activity of primary, human pulmonary artery endothelial cells (HPAECs) and on the endothelial monolayer integrity. We chose AuNPs as a standard due to their well-characterized optical and photothermal properties^30,31^, as well as our ability to tune their size, shape, and surface chemistry. Outlining a bovine serum albumin (BSA) pretreatment protocol, we developed pre-formed protein coronas to stabilize AuNPs in biological media. Overall, this study compares the effects of NP surface coating, size, and dose on the metabolic activity, cytoskeletal organization, and morphology of pulmonary endothelial cells.

## Results and discussion

### AuNP characterization

**Figure 1:**
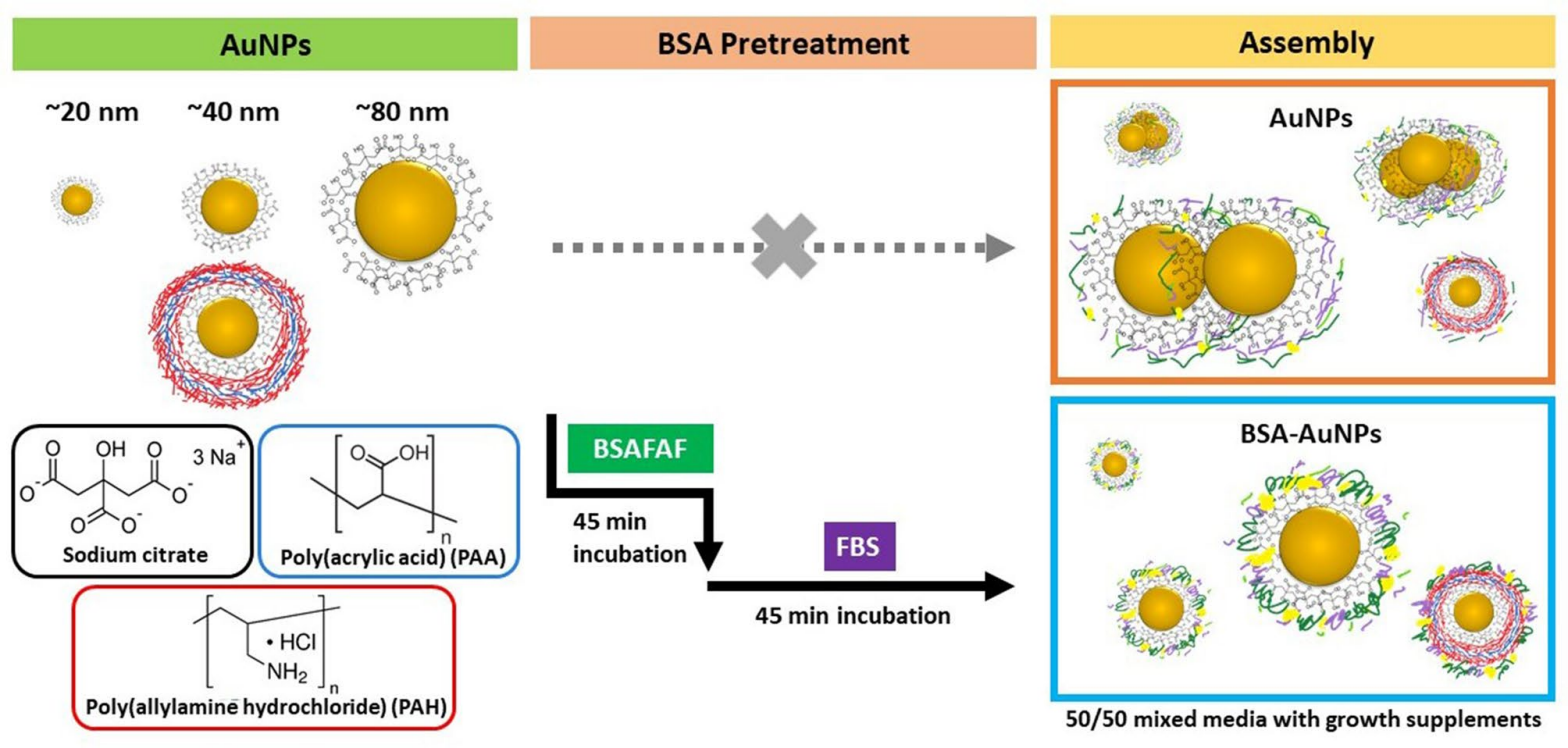
AuNP library of six different NPs. The library consists of three AuNP sizes with two surface coatings. AuNPs were treated with and without bovine serum albumin (BSA). AuNPs had diameters of ~ 20, 40, and 80 nm and surface coatings of citrate (black), and PAH (red). Pretreatment with bovine serum albumin-fatty acid free (BSA-FAF) followed by fetal bovine serum (FBS) improves the particles stability in the cell culture medium.

We built a AuNP library to study how AuNP dose, size, and surface chemistry affect the metabolic activity and morphology of HPAECs. We investigated three AuNP sizes with either of two surface coatings, citrate or poly(allylamine hydrochloride) (PAH) (Fig. 1). We then compared the effects of BSA pretreatment of NPs on endothelial cell metabolic activity and morphology.

**Figure 2:**
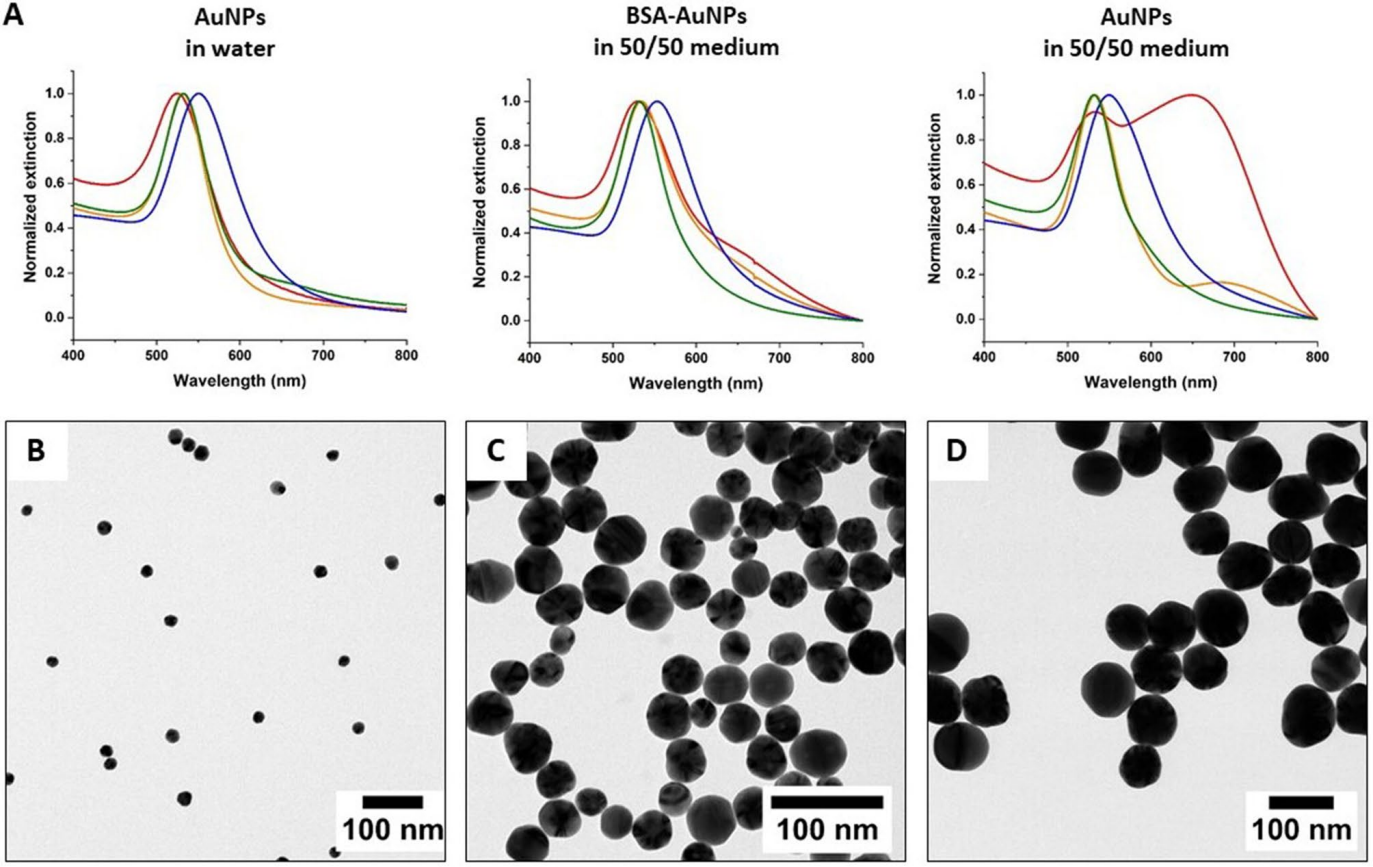
AuNP characterization by spectrophotometry and transmission electron microscopy. (**A**) UV–Vis–NIR spectra of four AuNPs in water, BSA-AuNPs in 50/50 medium, and AuNPs in 50/50 medium, respectively. The red, orange, green, and blue spectra correspond to citrate-AuNPs of 18 nm and 40 nm, PAH-AuNPs of 40 nm and citrate-AuNP 80 nm, respectively. Spectra were taken after AuNPs were incubated in water or in 50/50 medium for 1 h. TEM images of citrate-AuNPs of average diameters (**B**) 18 nm, (**C**) 40 nm, and (**D**) 80 nm.

After synthesis, a broad range of methods was used to characterize the NPs in aqueous solutions and in cell culture medium. Data were collected from AuNPs in water and AuNPs in 50/50 medium with and without BSA pretreatment. UV–Vis-NIR spectra of citrate-coated AuNPs (citrate-AuNPs) and triple-coated PAH AuNPs (PAH-AuNPs) are shown in Fig. 2a. Figure 2b,c,d shows the transmission electron microscopy (TEM) images of citrate-AuNPs with average diameters of 18 ± 3, 40 ± 11 or 80 ± 10 nm (Table 1). The surface chemistry of 40 nm citrate-AuNPs was modified using layer-by-layer (LBL) polyelectrolyte wrapping, in order to form PAH-AuNPs. For each sample, the PAH surface modification was confirmed by the change in the zeta potential from negative to positive, by the increase in the hydrodynamic diameter, and by the shift of the plasmon peak in water (Fig. S1). PAH-AuNP and BSA-PAH-AuNP stability was only achieved at 40 nm.

**Table 1:** Physical properties of AuNPs. Parameters include anhydrous and hydrodynamic diameters, plasmon band maxima and overall surface charges of AuNPs in water and in 50/50 medium. Anhydrous hydrodynamic diameters were determined by TEM. N refers to the number of particles measured. PAH-AuNPs resulted from tripled-coating citrate-AuNPs by alternative, positively (PAH), negatively (PAA) and positively (PAH) charged polyelectrolytes. Hydrodynamic diameters were measured by dynamic light scattering (DLS). For characterization of BSA-AuNPs in the 50/50 medium, AuNPs were incubated with 1 or 5 v/v% of BSA-FAF and 1 v/v% of FBS and then suspended in the 50/50 medium. The hydrodynamic diameters of the 50/50 medium with additional 1 v/v% of BSA-FAF / 1 v/v% of FBS and 5 v/v% of BSA-FAF / 1 v/v% of FBS were 20.5 ± 0.1 and 17.5 ± 0.4 nm, respectively. The zeta potential of the 50/50 medium with additional 1 v/v% of BSA-FAF / 1 v/v% of FBS and 5 v/v% of BSA-FAF / 1 v/v% of FBS were − 8.8 ± 0.5 and − 9.4 ± 0.6 mV, respectively. The hydrodynamic diameter and zeta potential of the 50/50 medium were 13.9 ± 0.3 nm and − 8.3 ± 4.5 mV. All data were reported as mean ± standard deviation.

TEM diameter (nm)		18 ± 3 (N = 369)	40 ± 11 (N = 383)		80 ± 10 (N = 329)
Property	Treatment	Citrate	Citrate	PAH	Citrate
$\lambda_{\max } ({\text{nm}})$	AuNPs in water	522	531	533	547
	BSA-AuNPs in 50/50 medium	530	534	532	553
	AuNPs in 50/50 medium	649	533	549	531
DLS diameter (nm)	AuNPs in water	28.6 ± 0.3	53.2 ± 0.3	58.2 ± 0.3	78.4 ± 0.4
	BSA-AuNPs in 50/50 medium	37.7 ± 0.1	103.9 ± 0.8	116.5 ± 0.3	127 ± 1
	AuNPs in 50/50 medium	149 ± 4	130 ± 3	124 ± 3	161 ± 3
Zeta potential (mV)	AuNPs in water	− 25.0 ± 0.4	21 ± 1	37 ± 3	− 39 ± 1
	BSA-AuNPs in 50/50 medium	− 10.1 ± 0.8	− 10.4 ± 0.6	− 9 ± 1	− 9.5 ± 0.8
	AuNPs in 50/50 medium	− 9.5 ± 0.1	− 9.5 ± 0.3	− 9.1 ± 0.4	− 8.9 ± 0.4

To improve particle stability, all AuNPs were pretreated with BSA and fetal bovine serum (FBS), and then were suspended in 50/50 medium. The characterizations of NPs with different sizes were done in different concentrations in order to keep all NP absorbance of UV–Vis-NIR spectra the same. The concentrations used for 18, 40 and 80 nm of AuNPs were 0.80, 0.030, and 0.0063 nM, respectively. All NP characterizations, including hydrodynamic sizes, zeta potentials and UV–Vis-NIR spectra were collected by this way. Table 1 shows the hydrodynamic sizes and zeta potentials of AuNPs. After the AuNPs were added to 50/50 medium, their hydrodynamic sizes increased, and all AuNPs showed slightly negative zeta potentials, regardless of their initial surface charges. Without BSA pretreatment to stabilize NPs, citrate-AuNPs of 40 and 80 nm show different degrees of aggregation in the 50/50 medium. Among all AuNPs, citrate-AuNPs of 18 nm show the highest degree of aggregation. A four-fold increase of the average hydrodynamic size from 37.1 ± 0.1 nm to 149.2 ± 1.3 nm was found when directly exposed to the 50/50 medium. At constant absorbance/extinction, the 18 nm citrate-AuNP spheres are at the highest nanoparticle concentration, and therefore are most prone to aggregation (as judged by the broadening and red-shifting of the plasmon band peak). Moreover, Fig. 2a shows a significant redshift of the maximum plasmon peaks (> 100 nm), multiple peaks and peak broadening in the UV–Vis-NIR spectra, confirming the presence of larger aggregates. The presence of a new peak at 649 nm after citrate-AuNPs of 18 nm directly mixed with the 50/50 medium is due to the plasmonic coupling of neighboring particles forming the aggregates^32,33^. Compared to 18-nm NPs, citrate-AuNPs of 40 nm aggregated less when directly added to the 50/50 medium. Their average hydrodynamic size increased slightly from 103.9 ± 0.8 nm to 129.9 ± 2.5 nm without BSA pretreatment. There was a minor peak present in addition to the maximum plasmon band in the UV–Vis-NIR spectrum (Fig. 2a). Unlike citrate-AuNPs, no obvious aggregation was observed for PAH-AuNPs of 40 nm and citrate-AuNPs of 80 nm when directly exposed to the 50/50 medium. However, their average hydrodynamic sizes resulting from BSA pretreatment were smaller than those without treatment. The UV–Vis-NIR spectra of BSA pretreated PAH-AuNPs (BSA-PAH-AuNPs) of 40 nm and BSA pretreated citrate-AuNPs (BSA-citrate-AuNPs) of 80 nm show narrower plasmon peaks compared to the spectra of NPs without prior treatment (Fig. 2a). Based on these results, we conclude that the introduction of additional proteins to AuNPs before exposure to the 50/50 medium contributes to NP stabilization. This was evident from smaller average hydrodynamic sizes of AuNPs and single and narrower plasmon peaks of the UV–Vis-NIR spectra.

### AuNPs modestly affect cell metabolic activity

**Figure 3:**
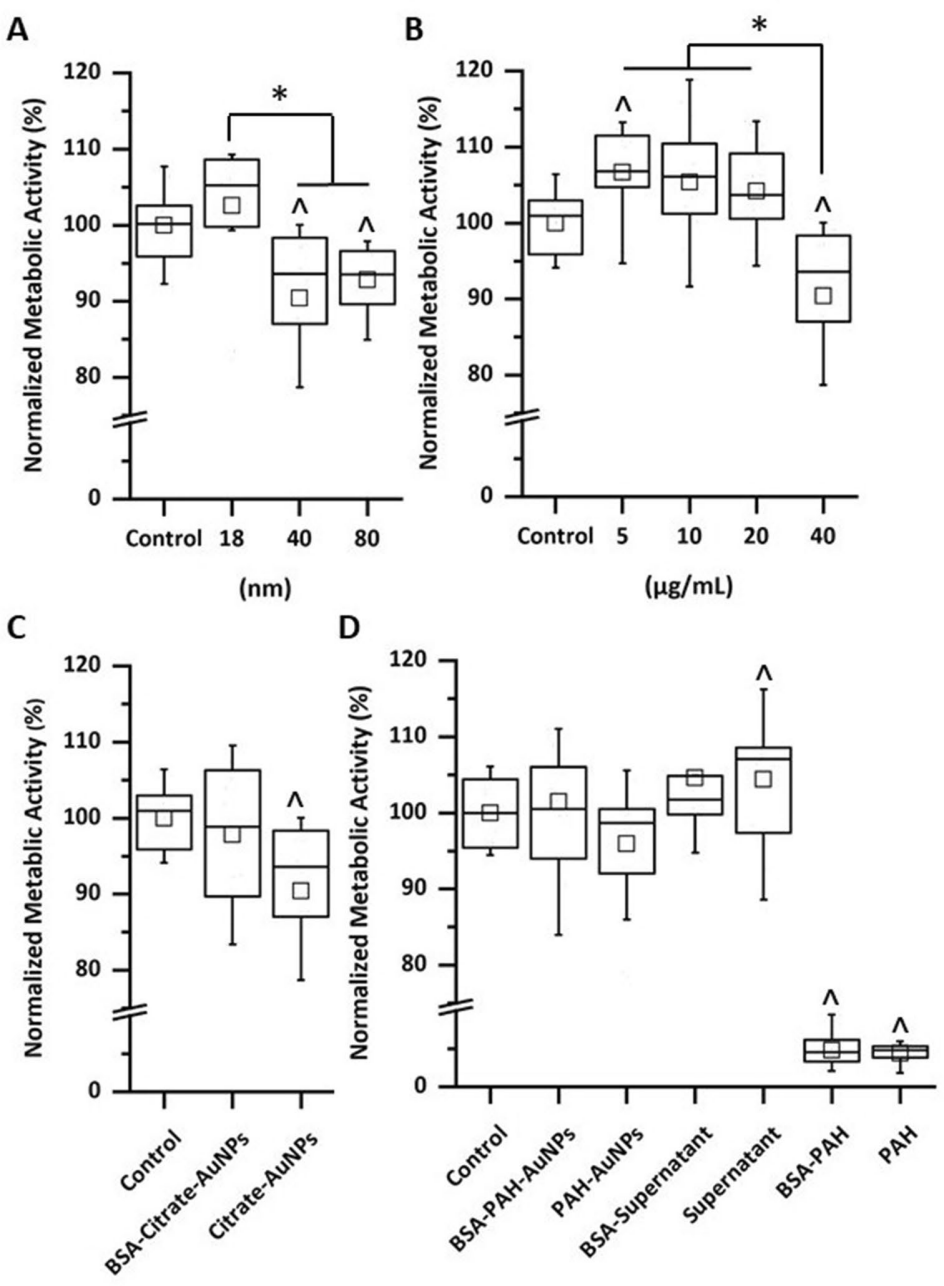
Normalized metabolic activity of cells after AuNP exposure. MTT assays of cell metabolic activity was determined after 18 h treatment with (**A**) citrate-AuNP (40 µg/mL) of 18, 40, and 80 nm, (**B**) 5–40 µg/mL of 40 nm, citrate-AuNPs, (**C**) 40 µg/mL of citrate-AuNPs (40 nm) with and without BSA pretreatment, and (**D**) 16 µg/mL of 40 nm, PAH-AuNPs and PAH-AuNP supernatant, and free PAH, all with and without BSA pretreatment. n = 8–19 wells per condition. The horizontal lines in the box plots indicate the median, boundaries of the box indicate the 25th and 75th percentile, whiskers include 95 percent of the data, and the square within box plot indicates average. Asterisks (*) indicate *p* < 0.05 and caret (^) symbols represent statistical significance compared to the control.

The effect of an 18 h AuNP exposure on the metabolic activity of HPAECs was determined with the MTT assay. As outlined in the Methods sections, background subtraction was utilized for all conditions to account for NP interference in the colorimetric MTT assay. Reported results are normalized to the control (no AuNP treatment). HPAECs were exposed to 40 µg/mL of citrate-AuNPs with diameters of 18, 40, and 80 nm. The 18 nm, citrate-AuNPs had no observed effect on HPAEC metabolic activity, compared to the control, with the metabolic activities being 102 ± 2% and 100 ± 1% respectively (Fig. 3a). Compared to untreated cells, exposure to 40 nm citrate-AuNPs slightly decreased metabolic activity, to 90 ± 3% (*p* = 9.2 × 10^−4^ relative to the control, KWANOVA). Exposure to 80 nm citrate-AuNPs decreased the metabolic activity to 92 ± 2% (*p* = 1.5 × 10^–3^ relative to the control, ANOVA). Many papers have shown size-dependent nanoparticle uptake, with 40–70 nm having higher rates of endocytosis than smaller particles, which may explain these results^34^. Overall, exposure to 40 and 80 nm citrate-AuNPs did have a statistically significant effect on HPAEC metabolism, but the decrease was modest, compared to the control.

To study how AuNP doses affect HPAEC metabolism, 5 to 40 µg/mL of 40 nm, citrate-AuNPs were used (Fig. 3b). At concentrations below 40 µg/mL, changes in metabolic activity after 18 h exposure were statistically not significant. However, exposure to 40 µg/mL of citrate-AuNP reduced the metabolic activity to 90 ± 3% (*p* = 7.9 × 10^–3^ relative to the control, KWANOVA). Exposure to 40 µg/mL of 40 nm, citrate-AuNPs—did have a statistically significant effect on HPAEC metabolism, but the decrease was modest, compared to the control.

Next, we studied the effect of surface chemistry and BSA pretreatment of 40 nm AuNPs on the HPAEC metabolic activity (Fig. 3c,d). Compared to the untreated control (100 ± 2%), 40 µg/mL of citrate-AuNPs decreased the metabolic activity to 90 ± 3% (*p* = 7.9 × 10^–3^ relative to the control, KWANOVA), while BSA-citrate-AuNPs decreased the metabolic activity only slightly to 97 ± 2%. BSA-PAH-AuNPs and PAH-AuNPs, at a concentration of 16 µg/mL, did not have statistically significant impacts on metabolic activity, with viabilities being 101 ± 3% and 96 ± 2%, respectively. We attribute the citrate-AuNP and PAH-AuNP modest impact on endothelial metabolic activity to the knowledge that bulk gold is chemically inert and safe at low doses^35^. Similarly, Liang et al. showed that treating human umbilical endothelial cells with 50 nm AuNPs did not decrease cell viability at low doses^18^. Overall, the data from HPAECs treated with citrate-AuNPs and PAH-AuNPs, with and without BSA pretreatment, suggest that 18 h AuNP treatment has modest effects on cell metabolic activity.

The LBL technique used to wrap the AuNPs requires an excess of polyelectrolytes; however, free polyelectrolytes can be cytotoxic^25,36,37^. We thus used centrifugation^38^ to remove the free polymers after coating the AuNPs. Following centrifugation, the amount of PAH in the supernatant of PAH-AuNP preparations was determined to be 0.4 ± 0.2 mg/mL. We tested the impact of the PAH-AuNP supernatant on metabolic activity. We also tested how the free polyelectrolytes affects cell metabolic activity, by exposing cells to solutions of comparable PAH concentrations. Similarly to the AuNPs, we treated the supernatant and the free PAH solution with the BSA pretreatment. Like the PAH-AuNPs, the supernatant had no impact on the HPAEC metabolic activity (Fig. 3d). When cells were treated with free PAH, with and without BSA treatment, a statistically significant decrease in the metabolic activity of HPAECs was observed: to 4.8 ± 0.6% (*p* = 6.7 × 10^–6^ relative to the control, KWANOVA) and to 4.5 ± 0.4% (*p* = 6.7 × 10^–6^ relative to the control, KWANOVA), respectively. In summary, although the PAH-AuNP supernatant had no impact on metabolic activity, the free PAH negatively impacted cell metabolism.

### AuNP exposure alters actin organization and disrupts cell–cell junctions

**Figure 4:**
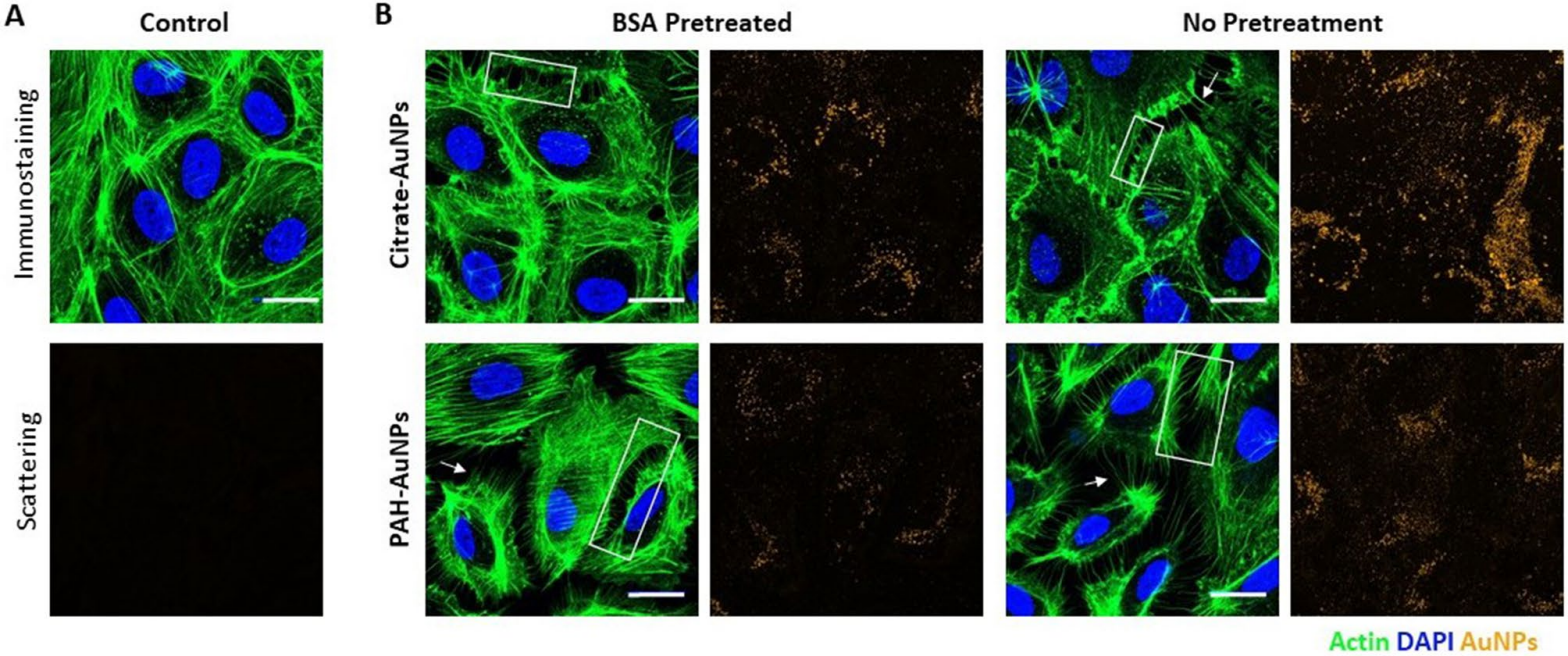
Comparison of untreated and BSA-pretreated AuNPs on actin (immunofluorescence) and particle uptake (light scattering) on HPAEC morphology. AuNPs were added directly to 50/50 medium or pretreated with BSA before addition to 50/50 medium. Immunofluorescence images of actin (green) and DAPI (blue) are shown for HPAECs at 18 h after treatment with AuNPs, with or without BSA. (**A**) Control with no AuNP treatment. (**B**) Exposure to Citrate- and PAH-AuNPs, with and without BSA pretreatment. Boxes indicate sites of inter endothelial gap formation. Arrows indicate radial actin fibers that are perpendicular to the boundaries of intercellular gaps. Representative images from each condition were chosen from n = 9–10 images. Images were obtained by a confocal fluorescence microscope and images were edited for clarity. Scale bar is 20 µm.

Next we investigated the changes in cell morphology upon exposure of HPAECs to 40 nm, citrate- (40 µg/mL) and PAH- (16 µg/mL) AuNPs, with and without BSA pretreatment. We chose to study the impact of 40 nm AuNPs on endothelial morphology because 40 nm AuNPs had the greatest impact on metabolic activity (vide supra). In addition, we established protocols for BSA pretreatment for 40 nm AuNPs. After 18 h of treatment with AuNPs, AuNP uptake correlated with cytoskeletal remodeling (Fig. 4). Compared to controls (Fig. 4a), AuNP exposure correlated with cortical actin remodeling (Fig. 4b). Instead of large actin fibers parallel to the junctions, the actin was more disorganized and exhibited more radial actin fibers perpendicular to cell boundaries. The actin remodeling also correlates with an increase in gap formation at cell–cell junctions. Radial actin fibers are apparent in regions adjacent to these gaps (Fig. 4b). The presence of these radial actin fibers is associated with increased cell contractility and higher tension on the junctions^39^. This cortical actin remodeling is more pronounced following PAH- and BSA-PAH-AuNP treatment, compared to citrate- and BSA-citrate-AuNP treatment. Taken together, our findings indicate that, although the different NP preparations used in this study only modestly alter metabolic activity of endothelial cells, AuNP exposure alters the actin organization and interendothelial integrity, as assessed by the radial actin fibers and prevalence of intercellular gaps. These results are in agreement with prior work by others. For example, Ma et al. reports human umbilical vein endothelial cell morphology changes induced by polymer-coated, anionic AuNP (Au-PMA NPs) treatment^18^. Similarly, Wang et al. report that negatively charged AuNPs target human microvascular endothelial cell junctions^40^. And Liu et al. conclude that changes in actin morphology following AuNP treatment result in human umbilical vein endothelial cell barrier dysfunction^19^.

**Figure 5:**
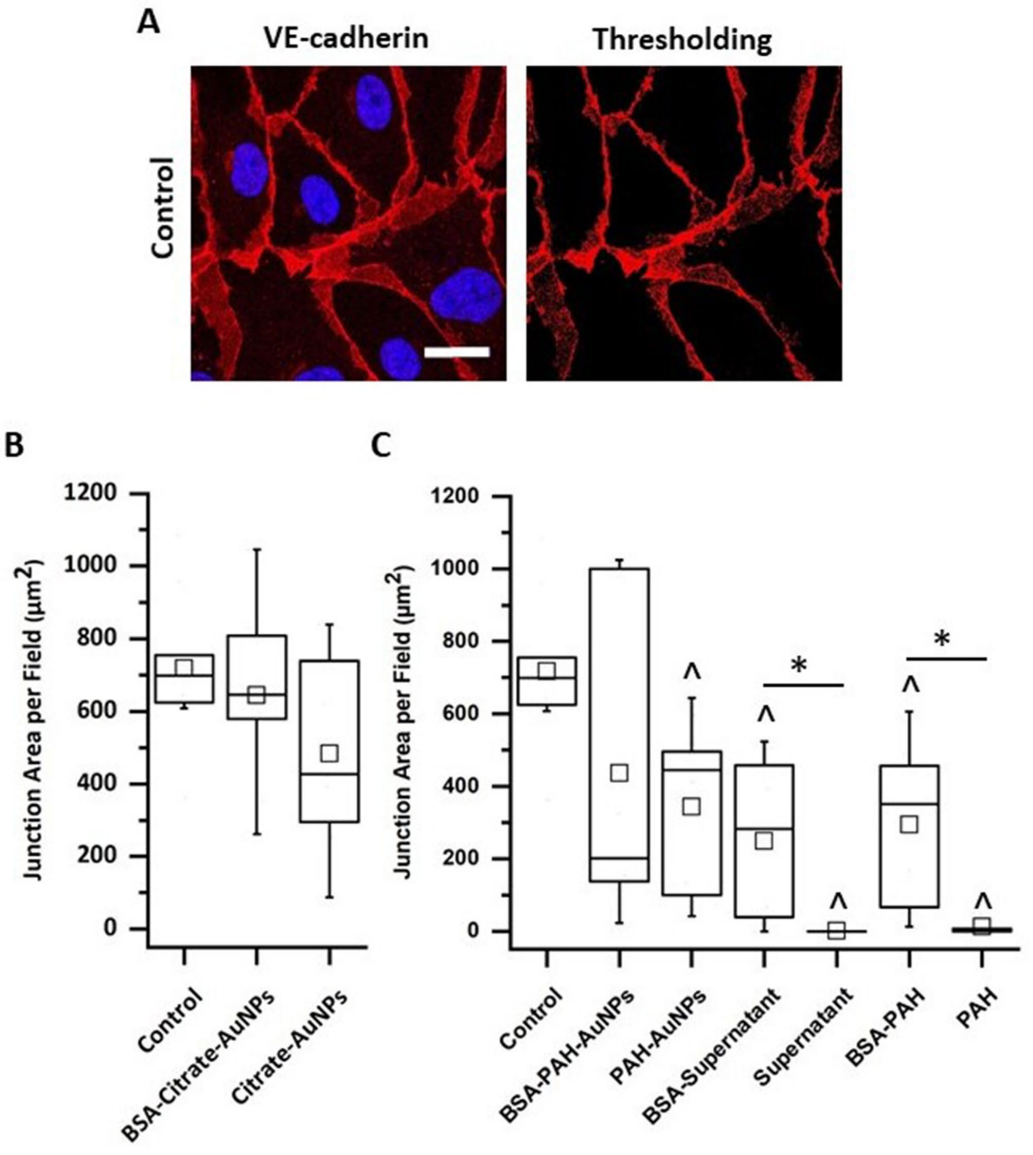
AuNPs alter the areas of inter endothelial junctions. HPAECs were treated with 40 µg/mL of citrate-AuNPs or 16 µg/mL of PAH-AuNPs in 50/50 medium for 18 h. (**A**) Confocal fluorescence image (left) of immunostained VE-cadherin (red) and nuclei (blue). Image processing based on a fluorescence intensity threshold was used to generate a mask that defines the area of the cell–cell junctions (right). Scale bar is 20 µm. HPAEC junction area per field determined following (**B**) citrate-AuNP treatment, with and without BSA pretreatment, and (**C**) PAH-AuNPs, PAH-AuNP supernatant, and free PAH treatment, with and without BSA pretreatment. n = 9–10 images per condition. The horizontal lines in the box plots indicate the median, boundaries of the box indicate the 25th and 75th percentile, whiskers include 95% of the data, and the square within box plot indicates average. Asterisks (*) indicate *p* < 0.05 and caret (^) symbols represent statistical significance compared to the control.

As mentioned in the Introduction, VE-cadherin is an intercellular adhesion molecule that localizes to cell–cell contacts and regulates actin organization at cell–cell junctions.^21,41^ VE-cadherin immunofluorescent images were used to quantify endothelial cell–cell junction area (Fig. 5a). Representative VE-cadherin staining and thresholding images for each condition used in Fig. 4 can be found in Fig. S2. HPAECs without AuNP treatment (control) had an average junction area of 720 ± 70 µm^2^ (Fig. 5b,c). Citrate-AuNP exposures, with and without BSA pretreatment, had no statistical impact on junction area compared to the control (Fig. 5b). BSA-PAH-AuNP exposure decreases the junction area to 400 ± 100 µm^2^^,^ but this is not a statistically significant reduction compared to the control (*p* = 0.18 against control, KWANOVA; Fig. 5c). Treatment of HPAECs with PAH-AuNPs led to a statistically significant decrease in junction area to 340 ± 80 µm^2^ (*p* = 7.5 × 10^–3^ relative to the control, ANOVA). This decrease in VE-cadherin area may indicate increased endothelial permeability^42,43^. Similarly, work by Setyawati et al. focused on the impact of titanium dioxide nanomaterials on human microvascular endothelial cells barrier leakiness by disrupting VE-cadherin interations.^22^ Other work has shown that barrier dysfunction induced by AuNPs is dependent on the NP size^20,44^. Our findings that PAH-AuNPs reduced pulmonary endothelial cell junction areas, indicative of reduced barrier function, are consistent with these other reports that AuNPs can increase vascular leakage.

The PAH-AuNP supernatant also induced a significant decrease in the junction area (Fig. 5c). Representative images of cell morphology following exposure to either PAH-AuNP supernatant or to free PAH are in Fig. S3. The BSA-supernatant also reduced the junction area to 250 ± 70 µm^2^ (*p* = 7.3 × 10^–4^ relative to the control, ANOVA), while treatment with supernatant reduced the junction area to 1 ± 1 µm^2^ (*p* = 3.3 × 10^–4^ relative to the control, KWANOVA) and thus completely disrupted the cell monolayer. Similarly, the HPAECs treated with the BSA-PAH exhibited a decrease in junction area to 300 ± 70 µm^2^ (*p* = 7.2 × 10^–4^ relative to the control, ANOVA), while the PAH treatment resulted in a decrease in junction area to 13 ± 7 µm^2^ (*p* = 3.2 × 10^–4^ relative to the control, KWANOVA). Interestingly, the BSA pretreatment of both the supernatant and soluble PAH led to increased junction area per field, compared to untreated supernatant. This finding suggests that the formation of complexes between PAH and BSA-FAF/FBS proteins^45,46^ reduces the negative effects of free polyelectrolytes, and in particular of free PAH. Work by Ball et al. shows that PAH and BSA interactions are endothermic resulting in enthalpically driven binding and the formation of large coacervate complex structures^45^. Furthermore, excess cytotoxic polyelectrolyte (PAH) in the PAH-AuNP supernatant negatively impacts endothelial cells, but BSA pretreatment can mitigate those negative impacts by forming complexes with the otherwise toxic PAH in the PAH-AuNP supernatant.

## Conclusion

Our results show that exposure of primary vascular endothelial cells with citrate- or PAH-AuNPs results in cortical actin remodeling and an increase in intercellular gap formation. These morphological effects are more prevalent following PAH-AuNP treatment compared to citrate-AuNP treatment. With this we conclude that the morphological effects resulting from PAH-AuNP treatment are mainly attributed to excess polyelectrolyte capping agent present in the supernatant of the PAH-AuNPs, rather than to effects of the AuNPs itself. While AuNP treatments alter cell morphology on varying degrees, AuNPs only modestly affect endothelial metabolic activity. If we extrapolate to the organism level, our results suggest that exposure of the lungs with AuNPs may lead to undesired endothelial permeability from the circulatory system to surrounding lung tissue. However, the here-introduced BSA pretreatment is used to improve AuNP colloidal stability in biological cell medium, as well as to mitigate the negative effects of the polyelectrolytes on endothelial cells. An interesting avenue of research that we are currently pursuing is to determine the impact of AuNPs on the permeability of co-cultures of lung endothelial and differentiated epithelial cells.

## Methods

### Reagents

Chloroauric acid (HAuCl_4_ 3H_2_O, ≥ 99.9%), sodium citrate tribasic dihydrate (Na_3_Ct·2H_2_O, ≥ 99.0%) hydroquinone (≥ 99%), poly(allylamine hydrochloride) (PAH, average Mw∼17,500), poly(acrylic acid, sodium salt) solution (PAA, average Mw∼15,000, 35 wt% in water), fluorescamine (≥ 98%), fetal bovine serum (FBS), bovine serum albumin-fatty acid free (BSA-FAF, heat shock fraction, lyophilized powder, essentially fatty acid free, ≥ 98%), bovine serum albumin (BSA), Triton X-100, and 4’,6-diamidino-2-phenylindole, dihydrochloride (DAPI) were purchased from Sigma-Aldrich (St. Louis, MO, USA). Sodium chloride (ACS grade) was acquired from EMD Chemicals (Branchburg, NJ, USA). Acetonitrile (≥ 99.9%) and hydrochloric acid were purchased from Fisher Chemical (Portsmouth, NH, USA). 3-(4,5-dimethylthiazol-2-yl)-2,5-diphenyltetrazolium bromide (MTT assay), 48-well BioLite plate, Dulbecco’s Modified Eagle Medium phenol-red-free (DMEM), Rhodamine Phalloidin, and ProLong Gold antifade mountant-was purchased from Thermo Fisher Scientific (Waltham, MA, USA). Human pulmonary artery endothelial cells and endothelial cell growth medium-2 (EGM-2) were from Lonza (Salisburg, MD, USA), while small airway differentiation medium (PneumaCult^TM^-ALI) was purchased from Stemcell Technologies (Seattle, WA, USA). Immunostaining antibodies vascular endothelial (VE)-cadherin (C-19) goat polyclonal IgG antibody, donkey anti-goat IgG-CFL 647 were from Santa Cruz Biotechnology, Inc (Dallas, TX, USA). Phosphate buffered saline (PBS) solution and 96 well plate (Corning Inc., Corning, NY, USA), proteomics grade sodium dodecyl sulfate (VWR, Radnor, PA, USA), #1.5 German cover glass (San Diego, CA, Cell E&G, USA), and #1 Micro coverglass—12 mm diameter (Electron Microscopy Sciences, Hatfield, PA, USA) were obtained from their respective manufacturers. All chemicals were used as received without further purification. Glassware was cleaned by aqua regia (a mixture of nitric and hydrochloric acids with a molar ratio of 1:3) and then water prior to synthesis. Water with a resistivity of 18 MΩ * cm (ultrapure water) was used for NP solution preparation and synthesis.

### Synthesis of AuNPs (~ 20 nm)

Citrate-AuNPs with diameters of ~ 18 nm were prepared by the Turkevich method^47^. First, 10 mL of 0.010 M of HAuCl_4_ 3H_2_O was added to 0.39 L of ultrapure water. The solution was stirred and heated to boiling on a hot plate. Once the solution was boiling, 8.0 mL of 5.0 wt% sodium citrate was introduced to the solution. Then the temperature of the solution was decreased to prevent further boiling; the solution was continuously heated for 30 min. After 30 min, 0.50 mL of 5.0 wt% sodium citrate was added to the solution and the solution was kept heated for an additional 10 min. To prevent particle aggregation due to an abrupt decrease in temperature, the solution was cooled naturally on the hot plate after the heat was turned off. Finally, the solution was centrifuged at 8,000×g for 20 min to remove excess citrate. The supernatant was removed, and the pellet was dispersed in ultrapure water.

### Synthesis of AuNPs (~ 40 and ~ 80 nm)

Larger AuNPs were synthesized by a seed-mediated growth method developed by Perrault and Chan.^48^ A gold seed solution was prepared by citrate reduction. First, a gold solution containing 0.12 L of ultrapure water and 1.2 mL of 1.0% (w/v) HAuCl_4_·3H_2_O was prepared. Next, the solution was stirred and brought to a rolling boil. Once the solution was boiling, 3.6 mL of 1.0 wt% sodium citrate was added. The temperature of the solution was reduced to prevent further boiling, but the solution was continuously heated for an additional 10 min. After 10 min, the heat was turned off and the solution was cooled naturally to room temperature (~ 1 h). To purify the AuNP seeds, the solution was centrifuged at 11,000×*g* for 20 min. The supernatant was removed, and the pellet was dispersed in ultrapure water.

To make hydroquinone-reduced AuNPs with diameters of ~ 40 and ~ 80 nm, aqueous stock solutions of 1.0% (w/v) HAuCl_4_·3H_2_O, and 0.030 M hydroquinone were freshly prepared. First, 1.0% (w/v) HAuCl_4_ 3H_2_O was centrifuged at 18,000×*g* for 60 min to remove any aggregates present in the solution. The supernatant was collected for further use and the pellet was discarded. Next, a growth solution containing 10 mL of 1.0% (w/v) HAuCl_4_**·**3H_2_O and 0.95 L of ultrapure water was prepared, and the solution was stirred rapidly at room temperature. Hydroquinone-reduced AuNPs with diameters of ~ 40 and ~ 80 nm were obtained by varying the amount of gold seeds added to the growth solution. To synthesize ~ 40 nm diameter AuNPs, 36 mL of 1.7 nM of the gold seed solution, 2.2 mL of 1.0 wt% sodium citrate and 10 mL of 0.030 M of hydroquinone were added in sequence to the growth solution and the solution was kept stirred for ~ 40 min. After 40 min, the solution was centrifuged at 5,000×*g* for 20 min. The supernatant was removed, and the pellet was dispersed in ultrapure water. AuNPs of ~ 80 nm diameter were prepared by the same procedure described above, except for a few differences: 8.0 mL of 1.7 nM of the gold seed solution was added to the growth solution, the growth solution was stirred for 60 min, and the growth solution was centrifuged at 1,000×*g* for 20 min. All particles were passed through surfactant-free, 0.22-µm filters prior to cell studies.

### Polyelectrolyte coatings of AuNPs

Positively charged, PAH-AuNPs were prepared by our previous method with the following modifications.^49,50^ To deposit the first layer of PAH onto ~ 40 nm citrate-AuNPs, a solution of 0.010 M NaCl and 10 mg/mL of PAH (1:2 in volume) was prepared. For an 1 mL scale polyelectrolyte wrapping, 0.3 mL of the NaCl and PAH solution was added to 0.90 mL of 0.1 nM AuNPs and the solution was gently shaken for 2 h. Following the 2 h incubation, the solutions were centrifuged at 4,900×*g* for 20 min. The supernatant was removed, and the pellet was dispersed in 0.81 mL of ultrapure water. Then, the deposition of PAA was initiated by adding 0.54 mL of 0.010 M NaCl and 10 mg/mL of PAA (1:2 in volume) to 0.81 mL of AuNPs. The solution was gently shaken for 2 h. After 2 h incubation, the solution was centrifuged at 4,900x*g* for 12 min. The supernatant was removed, the pellet was dispersed in 0.90 mL of ultrapure water. The procedure was repeated to prepare the final PAH layer: 0.30 mL of the mixed NaCl and PAH solution was added to 0.90 mL of AuNPs and the solution was gently shaken for 2 h. After 2 h incubation, the solution was centrifuged twice at 4,900×*g* for 12 min twice. The supernatant was removed, the pellet was dispersed in ultrapure water. All particles were passed through surfactant-free, 0.22-µm filters prior to cellular studies.

### Quantification of free PAH in AuNP suspensions

The supernatant from the second run of centrifugation was used to quantify free PAH present in the 40 nm, PAH-AuNP suspension (synthesis described above) and served as a free polyelectrolyte control for cell studies. We modified the procedures developed by Qiu et al.^38^ to quantify the amount of free PAH in the AuNP suspension using a fluorescence assay. Fluorescamine, a non-fluorescent compound, produces fluorophores when reacting with primary amines. To establish a calibration curve to determine the amount of PAH present in the AuNP suspension, 0.1% (w/v) of fluorescamine in acetonitrile and an aqueous standard solution of 0.2 mg/mL of PAH were prepared. Aliquots of 20-100 µL of 0.2 mg/mL of PAH were added to a 96-well microplate and each well was diluted with ultrapure water to 120 µL. Then, 120 µL of ultrapure water was added to each well. The supernatant isolated from 40 nm PAH-AuNPs was diluted to 10 times by ultrapure water and 120 µL of the diluted supernatant was added to each well. All wells were prepared in triplicate. Next, 60 µL of 0.1% (w/v) of fluorescamine was added to each well. After 15 min incubation at room temperature, the fluorescence intensity was determined by a fluorescence plate reader with E_ex_/E_em_ = 425 nm/480 nm. We used ultrapure water as a negative control to correct the fluorescence intensity of the standard PAH solutions and the AuNP supernatant.

### Bovine serum albumin (BSA) pretreatment

To prevent destabilization of AuNPs in 50/50 medium (50% endothelial culture medium (EGM-2, 2% (v/v) FBS) and 50% epithelial differentiation medium (PneumaCult^TM^-ALI Complete Base Medium)), AuNPs were first incubated with bovine serum albumin-fatty acid free (BSA-FAF, 1.2% (w/v) in 20 mM HEPES buffer) at room temperature. Citrate-AuNPs were incubated with 1% (v/v) of BSA-FAF and PAH-AuNPs were incubated with 5% (v/v) of BSA-FAF. After 45 min incubation, 1% (v/v) of fetal bovine serum (FBS) was introduced to the solution and followed by another 45 min incubation. Finally, 50/50 medium was added to the incubated AuNPs to make the desired AuNP concentration. The mixture of AuNPs and 50/50 medium was used in the following experiments after at a least 1 h incubation. All solutions were used within the same day of preparation. This stabilization protocol worked for citrate-AuNPs of all sizes and tested for concentrations between 5 and 40 µg/mL. For 40 nm PAH-AuNPs, the stabilization protocol worked for concentrations between 5 and 16 µg/mL. Following BSA pretreatment, AuNPs, citrate-AuNPs, and PAH-AuNPs are abbreviated as BSA-AuNPs, BSA-citrate-AuNPs, and BSA-PAH-AuNPs.

### Characterization of AuNPs

Hydrodynamic diameters and zeta potentials of AuNPs were determined with a ZetaPALS Particle Size and Zeta Potential Analyzer (Brookhaven, USA). UV−Vis−NIR spectra were measured with a Cary 5000 UV−Vis−NIR spectrophotometer (Agilent Technologies, USA). TEM of AuNPs was collected by a JEOL 2010 LaB6 or a JEOL 2100 Cryo electron microscope (JEOL Ltd., Japan). Average core diameters of AuNPs were determined by ImageJ software (National Institutes of Health, USA). At least 300 particles were counted to determine the diameters of AuNPs. The fluorescence intensity of the fluorescamine assay was determined by Tecan Infinite *M1000* PRO multimode reader (Tecan Trading AG, Switzerland).

### Cell culture growth conditions

Primary HPAECs were cultured in complete endothelial cell growth medium EGM-2 with 10% (v/v) FBS in a humidified incubator at 37 °C with 5% CO_2_ and passaged every 2–3 days. HPAECs between passages 7–9 were used for all experiments.

Exposure of HPAECs to AuNPs was achieved by incubating cells with the medium consisting of a mixture of equal parts of endothelial culture medium (EGM-2, 2% (v/v) FBS) and epithelial differentiation medium (PneumaCult^TM^-ALI Complete Base Medium); referred to as 50/50 medium. 50/50 medium was chosen, instead of pure endothelial growth medium, because the BSA pretreatment protocols were developed for further AuNP barrier studies with co-cultures of lung endothelial and epithelial cells.

### Quantifying cell metabolic activity

The metabolic activity of endothelial cells following AuNP treatment was evaluated using an MTT assay^51^. HPAECs (passage 7–9) were seeded at a density of 20,000 cells/well in culture medium. On day 2 when HPAEC layer was visually confluent under Nikon Eclipse TS100 (Nikon, Melville, NY, USA) microscope, AuNPs were suspended in 50/50 medium at the desired concentration and incubated for 18 h. Treatment of 40 nm, citrate-AuNPs, with and without BSA pretreatment, was completed at a concentration of 40 µg/mL. Treatment of 40 nm, PAH-AuNPs and supernatant, with and without BSA pretreatment, was completed at a concentration of 16 µg/mL. For control conditions, HPAECs were incubated with 50/50 medium and no AuNPs. Following incubation cells were rinsed with PBS and replaced with 20 µL of MTT (5 mg/mL in PBS) and 180 µL phenol red-free DMEM with 10% (v/v) FBS. After 2 h of MTT incubation, the 20 µL of SDS solution (10% (w/v) SDS in 0.01 M HCl) was added to each well to solubilize the formazan dye and incubated again for 2 h. Background subtraction wells, with no formazan formation, were measure to account for metallic NP interference in colorimetric MTT assay. For AuNP background subtraction wells, cells were rinsed with PBS and replaced with 180 µL of phenol red-free DMEM with 10% FBS (v/v) after 18 h AuNP incubation. After 2 h cell medium incubation, 20 µL of MTT (5 mg/mL in PBS) and 20 µL of SDS solution (10% (w/v) SDS in 0.01 M HCl) was added to each well and incubated again for 2 h, in order to prevent formazan formation from occurring. Absorbance was measured with a microplate reader Tecan Infinite M200 Pro (Tecan Trading AG, Switzerland) at 570 nm, which is the peak absorbance for formazan, and 680 nm. The A570 was referenced to A680 for each individual sample (Eq. 1). To account for NP interference in colorimetric measurement, formazan-only absorbance signal was calculated by subtracting the contribution of the background subtraction samples (no formazan formation) from samples (formazan formation). The mean formazan signal for control conditions (no AuNP treatment) was used to normalize data.

1 $$ (A570 - A680)_{{for.only}}  = (A570 - A680)_{{for. + NP}}  - {\mkern 1mu} (A570 - A680)_{{no{\kern 1pt} for.{\kern 1pt}  + NP}}  $$

Each formazan formation condition had two independent experiments, 3–6 wells were taken per experiment, and 2 colorimetric measurements were taken from each well; therefore, a total of 12–20 data points were collected per formazan formation condition. Each no formazan formation condition had two independent experiments, 1–2 wells were taken per experiment, and 2 colorimetric measurements were taken from each well; therefore, a total of 4–8 data points were collected per no formazan formation condition. Control conditions had 2–4 independent experiments, 1–2 wells were taken per experiment, and 2 colorimetric measurements were taken from each well; therefore, a total of 8–16 data points were collected per control condition.

### Immunostaining and imaging

HPAECs (passage 7–9) were seeded in 35 mm glass bottom dishes with 13 mm wells and #1.5 German cover glass at a density of 60,000 cells/well in the 13 mm glass slide portion in EGM-2 with 10% (v/v) FBS. On day 2 when HPAEC layer was visually confluent under Nikon Eclipse TS100 (Nikon, Melville, NY, USA) microscope, all the wells were rinsed with PBS to remove dead cells or debris and treated with AuNPs suspended in 50/50 medium at the desired concentration. Treatment of 40 nm, citrate-AuNPs, with and without BSA pretreatment, was completed at a concentration of 40 µg/mL. Treatment of 40 nm, PAH-AuNPs and supernatant, with and without BSA pretreatment, was completed at a concentration of 16 µg/mL. The plates were incubated in a humidified incubator at 37 °C with 5% CO_2_ for 18 h. Immunostaining was performed according to conventional protocols. Samples were then fixed using 2% paraformaldehyde in PBS for 15 min at 37 °C. Cells were permeabilized by 0.1% (v/v) Triton X-100 in PBS for 10 min at 37 °C, and non-specific antibody binding blocked by 1% (w/v) BSA for 1 h. Primary antibody staining by anti-VE-cadherin (C-19) goat polyclonal IgG antibody (1:100 dilution) was performed for 1 h. Secondary antibody staining was performed with donkey anti-goat IgG-CFL 647 (1:200 dilution) to image VE-cadherin and Rhodamine Phalloidin (1:200 dilution) to image F-actin for 1 h. Samples were mounted with DAPI (1:1000 solution) in Fluoromount-G, covered with a glass coverslip, and were dried overnight and stored at 4 °C until imaging. Samples were imaged using a Multiphoton Confocal Microscope Zeiss 710 (Carl Zeiss AG, Oberkochen, Germany). All fluorescence images were taken using a 63× oil immersion objective with same intensity and exposure time. Fluorescence visualized immunostaining and the reflectance mode of the confocal microscope provided high scattering cross sections of the AuNPs (559–566 nm). When capturing each single layer image for analysis, the microscope was focused on the cross section with clear definition in VE-cadherin. The example of an image captured and used for analysis, compared to the corresponding z stack projection is presented in Fig. S4. Each condition had two independent experiments and 4–5 images were taken per experiment (dish); therefore, a total of 9–10 images were collected per condition.

### Quantifying the area of endothelial cell–cell junctions

Image analysis was performed with ImageJ software 1.52b (National Institute of Health, Bethesda, MD, USA). To define the area of endothelial junctions, VE-cadherin images (8-bit) were used. A rolling ball radius of 22 nm (100 px) was chosen to perform a background subtraction. In order to accurately define the junctions and exclude cytoplasmic background, the threshold was adjusted from 0–50. Any remaining fluorescent regions of the cytosol were cropped out manually, leaving behind only junctions. Junction areas (pixels) were then quantified using the ‘Analyze particles’ tool in ImageJ. This area was converted and reported as junction area per field (µm^2^). Each condition had two independent experiments and 4–5 images were taken per experiment (dish); therefore, a total of 9–10 images were collected per condition. Average number of nuclei per image for all conditions is presented Fig. S5.

### Statistical analysis

Statistical analyses were performed with OriginLab (OriginLab Corporation, Northampton, MA, USA). Normality Test was used to identify normally distributed data. Grubbs Test was used to identify and remove outliers in not normally distributed data. Statistical significance for normally distributed data was determined using a one-way ANOVA. Kruskal–Wallis ANOVA (KWANOVA) was used to determine significance for not normally distributed data. A significance level of α = 0.05 was chosen. All data are reported as mean ± standard error of the mean, unless otherwise stated. The number of independent experiments and replicates performed for quantifying cell metabolic activity, immunostaining and imaging, and quantifying the area of endothelial cell–cell junctions can be found in respective Methods sections.

## Supplementary information

Supplementary Information.

## References

[1] Sambhy V, MacBride MM, Peterson BR, Sen A (2006). Silver bromide nanoparticle/polymer composites: Dual action tunable antimicrobial materials. J. Am. Chem. Soc..

[2] Espitia PJP (2012). Zinc oxide nanoparticles: Synthesis, antimicrobial activity and food packaging applications. Food Bioprocess Technol.

[3] Tungittiplakorn W, Cohen C, Lion LW (2005). Engineered polymeric nanoparticles for bioremediation of hydrophobic contaminants. Environ. Sci. Technol..

[4] Cecchin I, Reddy KR, Thomé A, Tessaro EF, Schnaid F (2017). Nanobioremediation: Integration of nanoparticles and bioremediation for sustainable remediation of chlorinated organic contaminants in soils. Int. Biodeterior. Biodegrad..

[5] Liu Z, Ling XY, Su X, Lee JY (2004). Carbon-supported Pt and PtRu nanoparticles as catalysts for a direct methanol fuel cell. J. Phys. Chem. B.

[6] Fang B, Chaudhari NK, Kim M-S, Kim JH, Yu J-S (2009). Homogeneous deposition of platinum nanoparticles on carbon black for proton exchange membrane fuel cell. J. Am. Chem. Soc..

[7] McNamara K, Tofail SAM (2017). Nanoparticles in biomedical applications. Adv. Phys..

[8] Sun T (2014). Engineered nanoparticles for drug delivery in cancer therapy. Angew. Chem. Int. Ed..

[9] Kairdolf BA, Qian X, Nie S (2017). Bioconjugated nanoparticles for biosensing, in vivo imaging, and medical diagnostics. Anal. Chem..

[10] Falagan-Lotsch P, Grzincic EM, Murphy CJ (2017). New advances in nanotechnology-based diagnosis and therapeutics for breast cancer: An assessment of active-targeting inorganic nanoplatforms. Bioconjug. Chem..

[11] Dreaden EC, Alkilany AM, Huang X, Murphy CJ, El-Sayed MA (2012). The golden age: Gold nanoparticles for biomedicine. Chem. Soc. Rev..

[12] Miller MR (2017). Inhaled nanoparticles accumulate at sites of vascular disease. ACS Nano.

[13] Ray PC, Yu H, Fu PP (2009). Toxicity and environmental risks of nanomaterials: Challenges and future needs. J. Environ. Sci. Health Part C Environ. Carcinog Ecotoxicol. Rev..

[14] Donaldson K (2005). Combustion-derived nanoparticles: A review of their toxicology following inhalation exposure. Part Fibre Toxicol.

[15] Miller MR, Shaw CA, Langrish JP (2012). From particles to patients: oxidative stress and the cardiovascular effects of air pollution. Futur Cardiol.

[16] Zhang Y, Yang W-X (2016). Tight junction between endothelial cells: The interaction between nanoparticles and blood vessels. Beilstein J. Nanotechnol..

[17] Lin IC (2011). Interaction of densely polymer-coated gold nanoparticles with epithelial caco-2 monolayers. Biomacromol.

[18] Ma X (2017). Colloidal gold nanoparticles induce changes in cellular and subcellular morphology. ACS Nano.

[19] Liu Y, Yoo E, Han C, Mahler GJ, Doiron AL (2018). Endothelial barrier dysfunction induced by nanoparticle exposure through actin remodeling via caveolae/raft-regulated calcium signalling. NanoImpact.

[20] Setyawati MI, Tay CY, Bay BH, Leong DT (2017). Gold nanoparticles induced endothelial leakiness depends on particle size and endothelial cell origin. ACS Nano.

[21] Brieher WM, Yap AS (2013). Cadherin junctions and their cytoskeleton(s). Curr. Opin. Cell Biol..

[22] Setyawati MI (2013). Titanium dioxide nanomaterials cause endothelial cell leakiness by disrupting the homophilic interaction of VE–cadherin. Nat. Commun..

[23] Tee JK (2019). Nanoparticles' interactions with vasculature in diseases. Chem. Soc. Rev..

[24] Walkey CD, Chan WCW (2012). Understanding and controlling the interaction of nanomaterials with proteins in a physiological environment. Chem. Soc. Rev..

[25] Alkilany AM (2009). Cellular uptake and cytotoxicity of gold nanorods: Molecular origin of cytotoxicity and surface effects. Small.

[26] Cedervall T (2007). Understanding the nanoparticle–protein corona using methods to quantify exchange rates and affinities of proteins for nanoparticles. Proc. Natl. Acad. Sci..

[27] Yin H (2015). Reducing the cytotoxicity of ZnO nanoparticles by a pre-formed protein corona in a supplemented cell culture medium. RSC Adv..

[28] Moore TL (2015). Nanoparticle colloidal stability in cell culture media and impact on cellular interactions. Chem. Soc. Rev..

[29] Gebauer JS (2012). Impact of the nanoparticle-protein corona on colloidal stability and protein structure. Langmuir.

[30] Sardar R, Funston AM, Mulvaney P, Murray RW (2009). Gold nanoparticles: Past, present, and future. Langmuir.

[31] West JL, Halas NJ (2003). Engineered nanomaterials for biophotonics applications: Improving sensing, imaging, and therapeutics. Annu. Rev. Biomed. Eng..

[32] Ojea-Jiménez I, Puntes V (2009). Instability of cationic gold nanoparticle bioconjugates: The role of citrate ions. J. Am. Chem. Soc..

[33] Park J-W, Shumaker-Parry JS (2014). Structural study of citrate layers on gold nanoparticles: Role of intermolecular interactions in stabilizing nanoparticles. J. Am. Chem. Soc..

[34] Chithrani BD, Ghazani AA, Chan WCW (2006). Determining the size and shape dependence of gold nanoparticle uptake into mammalian cells. Nano Lett..

[35] Alkilany AM, Murphy CJ (2010). Toxicity and cellular uptake of gold nanoparticles: What we have learned so far?. J. Nanopart. Res..

[36] Mackiewicz, M. *et al. The Impact of Surface Ligands and Synthesis Method on the Toxicity of Glutathione-Coated Gold Nanoparticles*. Vol. 4 (2014). 10.3390/nano4020355 PMC4512953 26213631

[37] Martinez JS, Keller TCS, Schlenoff JB (2011). Cytotoxicity of free versus multilayered polyelectrolytes. Biomacromol.

[38] Qiu TA (2017). Quantification of free polyelectrolytes present in colloidal suspension, revealing a source of toxic responses for polyelectrolyte-wrapped gold nanoparticles. Anal. Chem..

[39] de Rooij J, Kerstens A, Danuser G, Schwartz MA, Waterman-Storer CM (2005). Integrin-dependent actomyosin contraction regulates epithelial cell scattering. J. Cell Biol..

[40] Wang J, Zhang L, Peng F, Shi X, Leong DT (2018). Targeting endothelial cell junctions with negatively charged gold nanoparticles. Chem. Mater..

[41] Hoelzle MK, Svitkina T (2012). The cytoskeletal mechanisms of cell-cell junction formation in endothelial cells. Mol. Biol. Cell.

[42] Mehta D, Malik AB (2006). Signaling mechanisms regulating endothelial permeability. Physiol. Rev..

[43] Birukova AA (2012). VE-cadherin trans-interactions modulate Rac activation and enhancement of lung endothelial barrier by iloprost. J. Cell. Physiol..

[44] Liu Y (2017). Nanoparticle size-specific actin rearrangement and barrier dysfunction of endothelial cells. Nanotoxicology.

[45] Ball V (2002). Complexation mechanism of bovine serum albumin and poly(allylamine hydrochloride). J. Phys. Chem. B.

[46] Becker AL, Henzler K, Welsch N, Ballauff M, Borisov O (2012). Proteins and polyelectrolytes: A charged relationship. Curr. Opin. Colloid Interface Sci..

[47] Frens G (1973). Controlled nucleation for the regulation of the particle size in monodisperse gold suspensions. Nat. Phys. Sci.

[48] Perrault SD, Chan WCW (2009). Synthesis and surface modification of highly monodispersed, spherical gold nanoparticles of 50–200 nm. J. Am. Chem. Soc..

[49] Gole A, Murphy CJ (2005). Polyelectrolyte-coated gold nanorods: Synthesis, characterization and immobilization. Chem. Mater..

[50] Dennison JM, Zupancic JM, Lin W, Dwyer JH, Murphy CJ (2017). Protein adsorption to charged gold nanospheres as a function of protein deformability. Langmuir.

[51] Mosmann T (1983). Rapid colorimetric assay for cellular growth and survival: Application to proliferation and cytotoxicity assays. J. Immunol. Methods.

