# Supplementary material for: Gold nanoparticles disrupt actin organization and pulmonary endothelial barriers
Source: Sci Rep. 2020 Aug 7;10:13320. doi: 10.1038/s41598-020-70148-1 (PMC7414109; doi:10.1038/s41598-020-70148-1)
Supplement: Supplementary file 1 — Supplementary Information. [file 41598_2020_70148_MOESM1_ESM.pdf]

Supporting Information

Gold nanoparticles disrupt actin organization and pulmonary endothelial barriers

Whitney E. Sinclair[1], Huei-Huei Chang[2], Arkaprava Dan[1], Paul J. A. Kenis[1,3], Catherine J. Murphy[2], and Deborah E. Leckband[1,2,3,*]

[1] University of Illinois at Urbana-Champaign, Department of Chemical and Biomolecular Engineering, Urbana, 61801, USA

[2] University of Illinois at Urbana-Champaign, Department of Chemistry, Urbana, 61801, USA

[3] University of Illinois at Urbana-Champaign, Carl Woese Institute for Genomic Biology, Urbana, 61801, USA

[*]leckband@illinois.edu

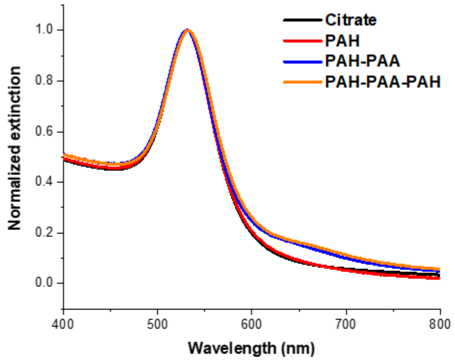

λ_{max} (nm)	Zeta potential (mV)	Dynamic light scattering (DLS) sizes (nm) in ultrapure water
531	-21 ± 1	53.2 ± 0.3
531	34 ± 4	N/A
531	-30 ± 4	N/A
533	37 ± 3	58.2 ± 0.3

Fig. S1: Changes of plasmon peaks, zeta potentials and hydrodynamic diameters in nanopure water after polyelectrolyte coatings of AuNPs. UV−vis−NIR spectra of AuNPs show the black, red, blue and orange spectra corresponding to citrate-, PAH- and PAA-PAH and triple-coated PAH-PAA-PAH AuNPs, respectively.

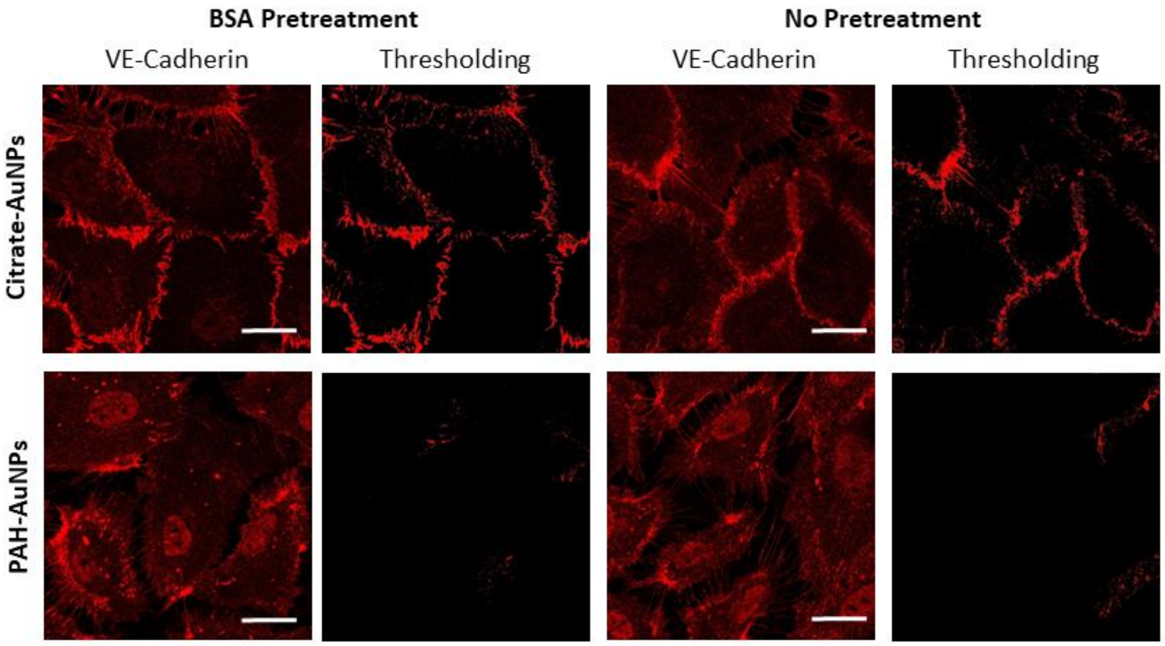

Fig. S2: HPAECs on glass bottom dishes were treated with AuNPs, with or without BSA pretreatment, and immunostained for VE-cadherin after 18 h. Image processing was used to develop a threshold of the VE-cadherin junction area per field. VE-cadherin images for citrate-AuNP and PAH-AuNP treatments correspond to morphological images in Figure 4. Images were obtained by a confocal fluorescence microscope with immunostained VE-cadherin (red). VE-cadherin immunostaining images were edited for clarity. Scale bar is 20 μm.

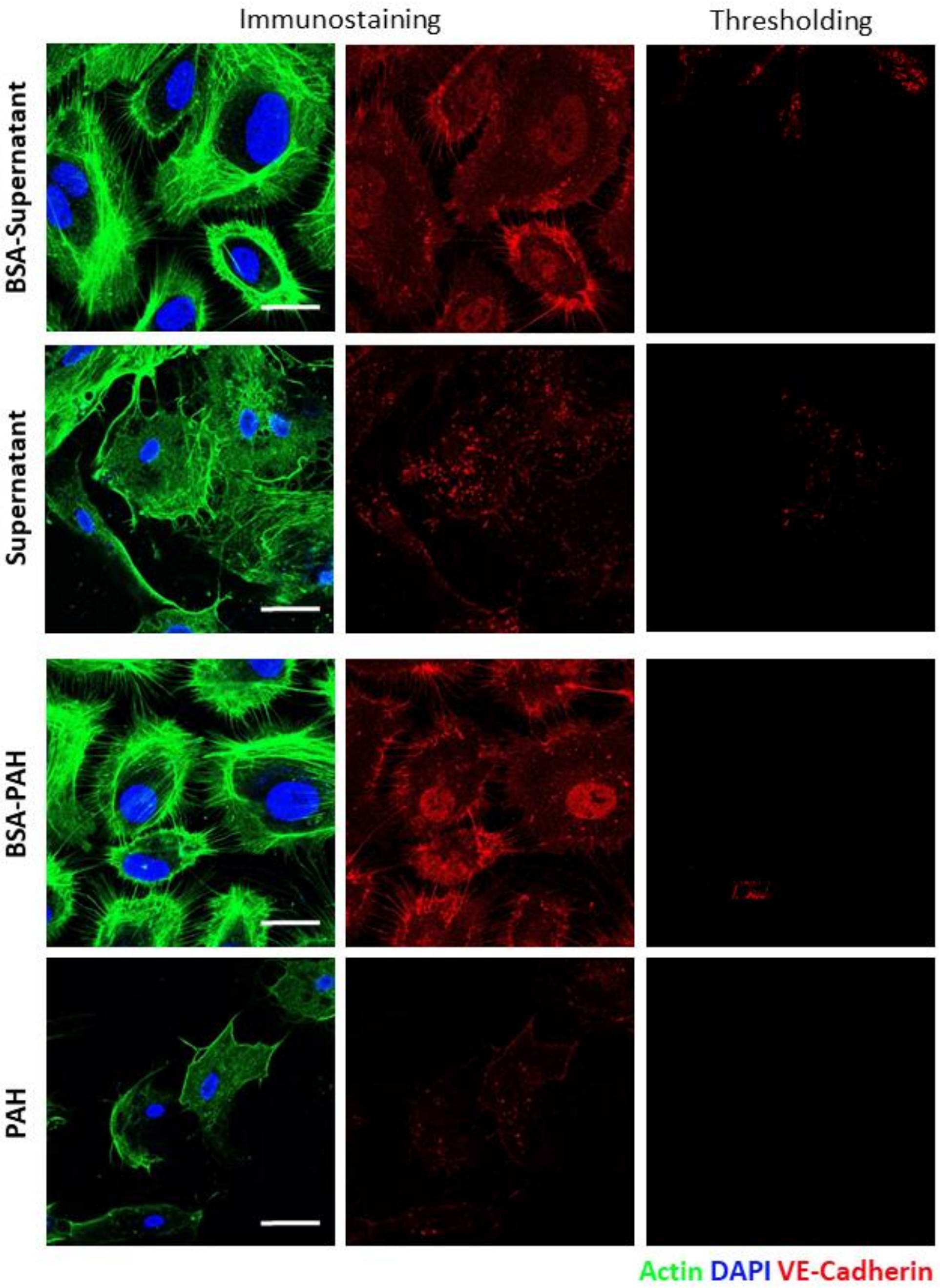

Fig. S3: HPAECs on glass bottom dishes were treated with supernatant or PAH, with or without BSA pretreatment, and immunostained for actin, nuclei, and VE-cadherin after 18 h. Image processing was used to develop a threshold of the VE-cadherin junction area per field. Images were obtained by a confocal fluorescence microscope with immunostained actin (green), nuclei (blue) and VE-cadherin (red). Immunostaining images were edited for clarity. Scale bar is 20 µm.

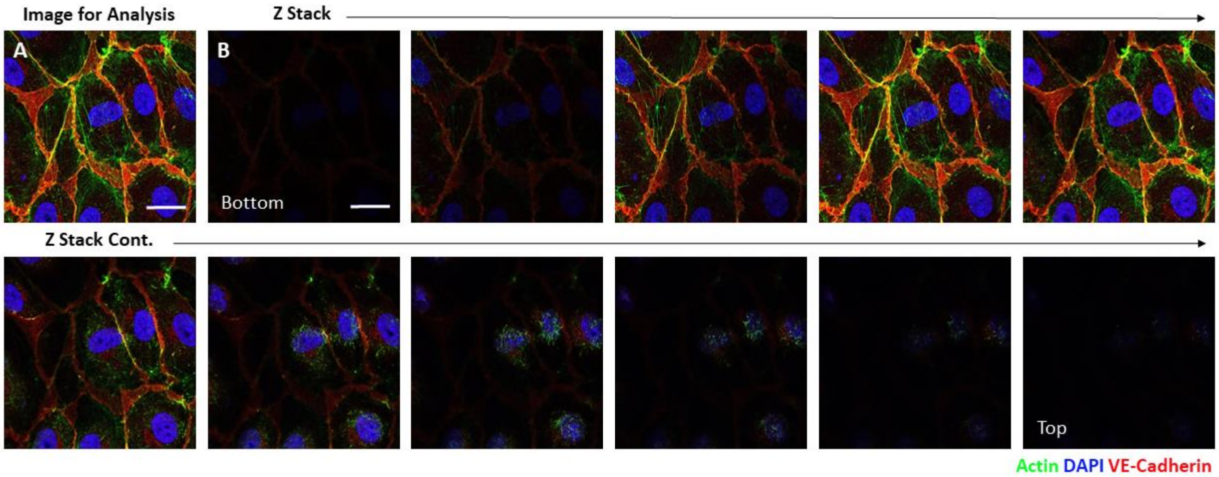

Fig. S4: HPAECs on glass bottom dishes was immunostained after 18 h with no AuNP treatment. Images were obtained by a confocal fluorescence microscope with immunostained actin (green), nuclei (blue) and VE-cadherin (red). (A) Image of single layer cross section that was used for image analysis. (B) ~5 µm z stack cross section of HPAECs with 0.5 µm step size. Immunostaining images were edited for clarity. Scale bar is 20 µm.

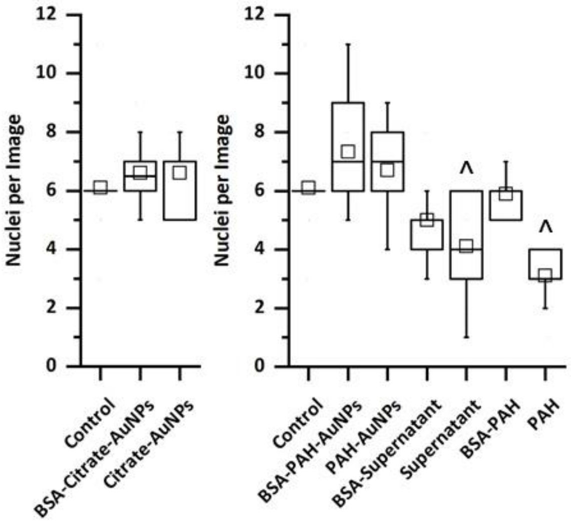

Fig. S5: Average number of nuclei per image for each treatment condition. Each condition had two replicates and 4-5 images were taken per replicate. n = 9-10 images per condition. The horizontal lines in the box plots indicate the median, boundaries of the box indicate the 25th and 75th percentile, whiskers include 95 percent of the data, and the square within box plot indicates average. Caret (^) symbols represent statistical significance ($p < 0.05$) compared to the control.
